# Why apple orchards are shifting to the higher altitudes of the Himalayas?

**DOI:** 10.1371/journal.pone.0235041

**Published:** 2020-07-10

**Authors:** Netrananda Sahu, Atul Saini, Swadhin K. Behera, Takahiro Sayama, Limonlisa Sahu, Van-Thanh-Van Nguyen, Kaoru Takara

**Affiliations:** 1 Department of Geography, Delhi School of Economics, University of Delhi, Delhi, India; 2 Disaster Prevention Research Institute, Innovative Disaster Prevention Technology and Policy Research Laboratory, Kyoto University, Gokasho, Uji City, Kyoto, Japan; 3 Application Laboratory, Japan Agency for Marine-Earth Science and Technology, Yokohama, Japan; 4 Department of Environmental Science, Fakir Mohan University, Odisha, India; 5 Department of Civil Engineering and Applied Mechanics, McGill University, Montreal, Canada; 6 Graduate School of Advanced Integrated Studies (GSAIS) in Human Survivability (Shishu-Kan), Kyoto University, Kyoto, Japan; Potsdam Institute for Climate Impact Research, GERMANY

## Abstract

Apple cultivation is one of the most important sources of livelihood in Indian side of the Himalayas. The present study focuses on the apple orchards of Himachal Pradesh, a state within the Himalayan Mountains, a major apple producers of India. In the study, it is found that the optimum apple growing conditions in the region have been consistently shifting and farmers are shifting their orchards to the higher altitudes. For example, orchards have shifted to 1500–2500 meters in the 2000s compared to the cultivated elevation of 1200–1500 meters during 1980s. As of 2014, apples are being cultivated at an elevation of more than 3500 meters, for example, the newly developed orchards of Leo village in upper Kinnaur and Keylong area of Lahul and Spiti districts. Chilling hours for different districts are calculated. The trend of temperature during the growth period, winter session and annual rainfall have been analysed using Mann-Kendall and Sen’s slope test. Data catalogued from different time periods indicates that the northward shift (towards higher altitude) is due to changes in chilling hours, total annual rainfall and mean surface temperature during the apple growing season. The mean surface temperature in all the districts has increased by almost 0.5°C during last 2000–2014. These changes are directly related to global warming. While the changing climate is reducing the apple production in low altitudinal regions of the state, it is creating new opportunities for apple cultivation in higher altitudes as conditions are getting more favourable for apple growth in those higher regions. The associated socio-economic changes are posing new societal issues for the local farmers.

## 1. Introduction

Situated in north-western Himalayas, Himachal Pradesh is the mountainous state of India where apple cultivation is one of the most popular farming activities. Though the variation in topography inundated with mountains and valleys attributes differential conditions and agricultural practices, apple cultivation remains as a major source of livelihood and means of financial security for the people of the state [1]. The state contributes to a large majority of the total national apple production, particularly the high-quality ones in India [2]. According to the National Horticulture Board of India, the ideal climate conditions for apple cultivation in Himachal Pradesh of India include a mean temperature of 21°C - 24°C during growth period of six months from March-August (MAMJJA), total annual rainfall of 100–125 cm, and 1000–1500 chilling hours [3, 4]. Chilling hours are defined as the number of hours in which temperature remains less than 7°C. Chilling hours is one of the very important conditions for the growth of apple and any changes in those optimum climate conditions lead to adverse growth of apple and hence influence the apple production [5]. Less chilling hours mainly lead to abnormal flowering and blooming, which results in a high probability of dormancy in apple buds [6].

In Himachal Pradesh, apples were known to be cultivated at altitudes between 1200–1500 meters in the early 1980s [4]. Orchards have shifted to 1500–2500 meters in the 2000s. More recently apples are now being cultivated at more than 3500 meters elevation in the Himachal Pradesh, owing to warming of the surface temperature since the 1980s [7]. Consequently, there was notable difference in production. Changes in climatic conditions have led to the appearance of diseases in apple like scub diseases, premature defoliation, alternaria, and alternaria iternate. These diseases sometimes become endemic when the climatic condition favours an optimum window for the disease proliferation [8]. Similarly, sensitivity of the apple to spring frost is analysed and it gives a critical idea on loss to apple orchard with late or early spring frost event in warmer climate conditions [9]. Moreover, global warming is reported to have played an important role in changing the texture and taste of the fruit [10]. With the rise in surface temperature, apples orchards have consistently been relocated to higher altitudes to retain the optimal conditions for the fruit growth.

However, while it is known that these orchards are changing locations, exactly what climatic variables are responsible for these changes are yet not well-documented. It is also not clear how these climatic factors are influencing the decision-making processes of the farmers, who choose to relocate their orchards, and the associated socio-economic impacts. It should be noted that availability of new lands for apple cultivation at higher altitudes has encouraged new farmers from those areas to start apple cultivations. In this manner the farmers at higher altitudes are getting benefitted by natural availability of optimum chilling hours. Though some farmers at previously apple-growing lower-altitudes try to shift their orchards to the higher altitudes most of them have switched to the cultivations of other profitable crops like mushrooms and vegetables for their livelihood.

The goal of this paper is to study the key factors, especially the climate processes, responsible for shifting of apple orchards to the high altitudinal region of Himachal Pradesh by analysing the changes in important climate variables. Though previous studies have suggested the climate change to be a factor in the altitudinal shifting of apple orchards [11–18] the exact climate processes responsible for the decrease in cultivated areas and apple production in lower altitudes of Himachal Pradesh are not explored. In this study, we investigated different climatic parameters i.e. minimum, mean and maximum surface temperature variations, chilling hours, rainfall pattern, and attempted to find the link with apple cultivated area and apple productions from 1975–2014 in the state of Himachal Pradesh under the climate change scenarios to achieve the objective of the study.

## 2. Data and methods

### 2.1 Study area

**Fig 1:**
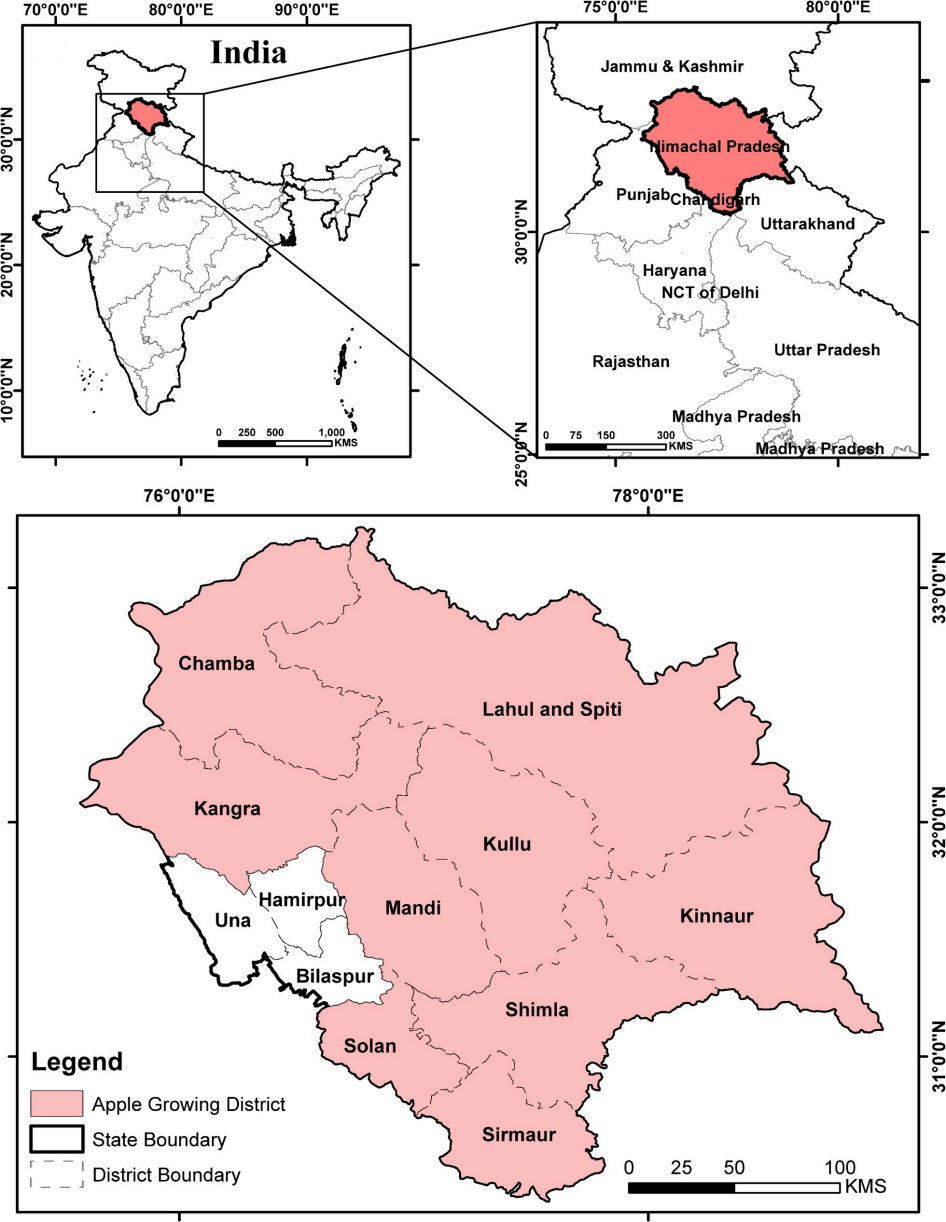
Location of Himachal Pradesh in India and all the 9 apple growing districts of the state. All the districts except Hamirpur, Una and Bilaspur are the part of present research study.

Himachal Pradesh is a mountainous state of India and located in the Himalayas. Administratively it is divided into 12 districts and only 9 districts of Himachal Pradesh are suitable for apple productions (Fig 1). Due to its location in the high Himalayas, the state observes very low temperature during winter seasons in comparison to the neighbouring riparian states. Therefore, occurrence of required number of chilling hours for apple cultivation make this state suitable for its cultivation.

Moreover, shifting of apple orchards to the higher altitudes imposes a threat to the low altitude districts of Kangra, Sirmaur and Solan in the near future. We classified the apple cultivation districts of Himachal Pradesh based on altitude into three categories (a) high altitudinal (Lahul & Spiti, Kinnaur and Chamba, ≈ 2500 -3500m and above) (b) mid altitudinal (Shimla, Kullu, Mandi and Kangra, ≈1500–2500 m) and (c) low altitudinal (Solan and Sirmaur, ≈1000-1500m). Chilling hours were calculated because of its importance for the growth of apples [11–13], as it influence the apple bud strength to break the dormancy.

### 2.2 Methodology

In order to assess climatic changes impacts on causing the shift of optimum conditions to higher altitudes, we focused on the necessary conditions for the apple cultivations as suggested by the National Horticulture Board of India. We have examined surface temperature, rainfall, chilling hours, cultivation area, and apple productivity for this study. Additionally, apple thrive when the plants are cultivated in loamy soil with pH values of 5.5–6.5. However, pH observations were not considered in the present study as Himachal Pradesh is part of the Himalayan range and is well known for ideal soil conditions for apple.

#### 2.2.1 Statistical techniques

Linear regression method is used to analyse the climate variables (temperature and rainfall) with apple area and production. District wise trend of surface temperature and rainfall was also analysed. The trend of surface temperature, rainfall, chilling hours, apple production and area were investigated using the Mann-Kendall [19] and Sen’s slope [20, 21] for the period 1975–2014. The selection of the period 2000–2014 for the detailed analysis is based on the results of highly intense change in the production of apple in the high altitudinal districts.

To identify the intensity of change point, we used the ‘strucchange’ package in R-programme and the least square in the leaner trend is obtained for production and area. The obtained point of time (i.e. year) in production and area trend from 1975–2014 for Lahul & Spiti, Kinnaur and Chamba are 2006,2002 and 2007 and 1996,2004 and 2009 respectively [22–24]. The majority of change point year for production and area of all the three districts were lying in the period later to the year 2000 and the calculated average breakpoint year was 2004. Following the identification of the change point in the time series, the intensity of change in surface temperature and rainfall for the recent past (2000–2014) was done using the base period 1951–2000. Moreover, due to availability of data from 1975–2014, the intensity of change of apple production and cultivation area for 2000–2014 period was estimated using the mean values for the base period 1975–2000.

#### 2.2.2 Calculation of chilling hours

Chilling hours were calculated by using the model of Dale. E. Linvill, which is also known as Modified UTAH model [25] and for its calculation we obtained the temperature data from the National Climate Centre, India Meteorological Department, Government of India [26]. To calculate the daytime and night time chilling hours, day length was obtained as a pre-requisite for chilling hours. Subsequently, the sum of daytime and night time chilling hours contributed to daily chilling hours. For whole Himachal Pradesh, chilling hours persist from December to February (DJF), which is the winter season. Therefore, those three winter months were considered for the calculation of the chilling hours.

**Fig 2:**
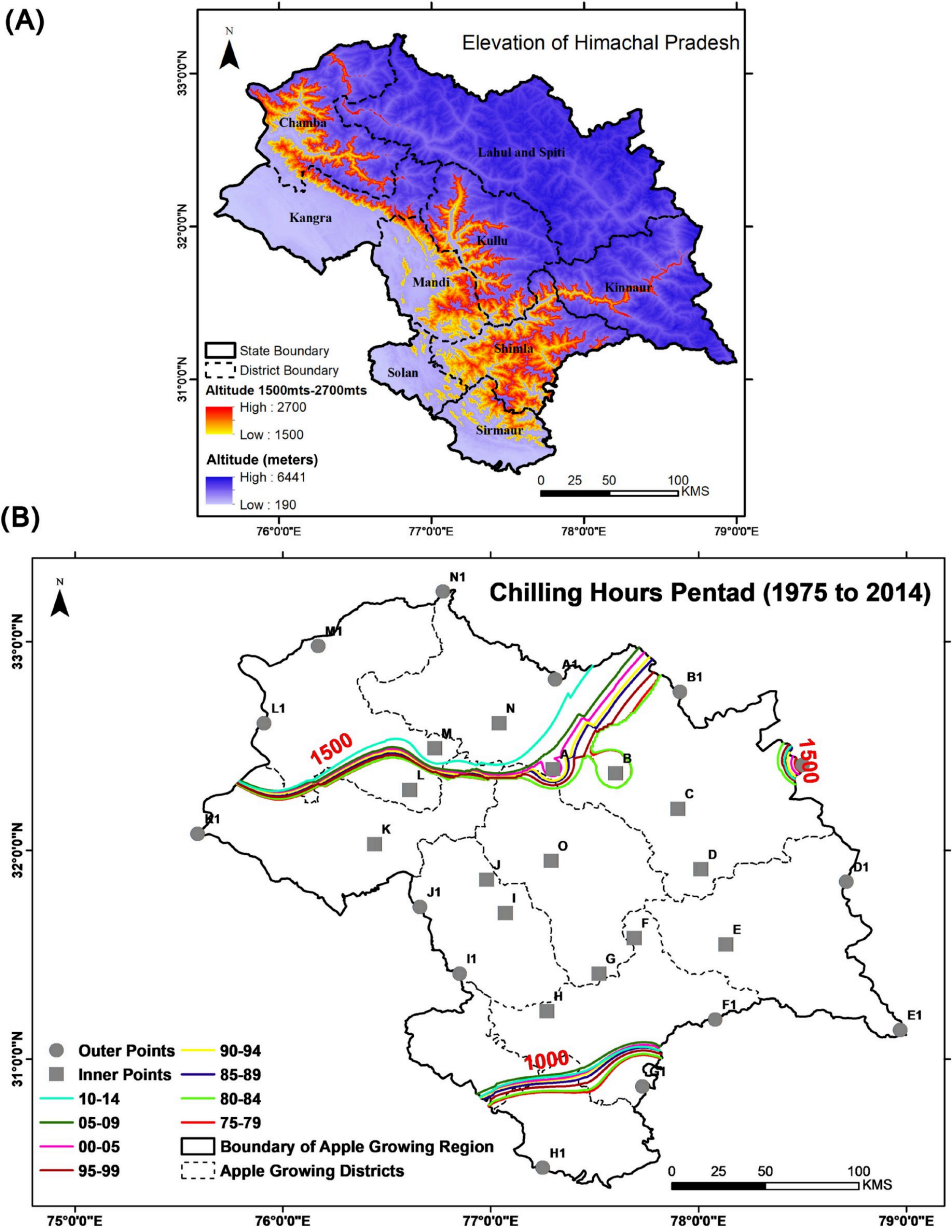
Panel (A) Elevation of Himachal Pradesh shown using Cartosat-DEM and as per the standards, the altitude suitable for apple cultivation is highlighted in shades of red colour; Panel (B) Shifting of the chilling hours from the period 1975–1979 to 2010–2014. Grey colour filled circles and squares show the points considered to extract temperature value for calculating chilling hours. Lines with different colours represent the optimum chilling hours for a particular period as given in the legend.

Seasonal warming terminates the chilling hours in the later seasons. To calculate pan-Himachal Pradesh chilling hours (Fig 2A), a centroid of apple growing districts was derived (‘O’ in the Fig 2B). Following which other points of proximity to the centroid were chosen randomly (‘A’ to ‘N’) and that led to determine all secondary points (Fig 2B) on the border (‘A1’ to ‘N1’). Temperature values at all these points helped us to calculate the chilling hours from 1975 to 2014. Adopted chilling hour model of Dale E. Linvill [25] in the present study is as follows:

DL=12.14+3.34×tan(LA)×cos(0.0172×CD−1.95) (1)

$$CH=\left[\frac{\left(DL+4\right)}{\pi }\right]\times arcsin\left[\frac{\left(Tc-Tmin\right)}{\left(Tmax-Tmin\right)}\right]$$ (2)

$$CH=(24-DL)-exp\left\{[\frac{\left(Tc-Ts\right)}{\left(Ts-Tmin\right)}]\times ln(24-DL)\right\}$$ (3)

Where CH = chilling hour, DL = Day length, CD = Climatological day, LA = Latitude, T_C_ = Critical temperature (reaching the chilling hour), T_S_ = Temperature before sun rise and sun set for DL.

## 2.3 Data

### 2.3.1 Surface temperature and rainfall

Gridded rainfall dataset of spatial resolution (0.25° x0.25°) [26] and surface temperature data with spatial resolution (1°x1°) [27] of daily arithmetic mean were obtained from the National Climate Centre, India Meteorological Department, Government of India for the period of 1951–2014. The unit of rainfall in this study is millimetre (mm). The same temperature data was used in previous studies related to temperature in India [28, 29]. NCAR command language was used to make the maps, figures and tabulate the change of temperature and rainfall at different regional scales. Pentad maps of temperature were used to assess the change in mean temperature during the growth period and total annual rainfall.

#### 2.3.2 Apple cultivation area and production

Apple cultivation area and production data were obtained from the office of the Directorate of Horticulture, Himachal Pradesh located in the provincial capital Shimla. Directorate of Horticulture, Himachal Pradesh state implements horticulture-related programs and maintains horticulture data of the state. Using these data, the cultivation area and production percentages were calculated. Digital elevation model Cartosat-1 (Version-3 R1) was obtained from the *Bhuvan* online platform of the Indian Space Research Organization to get the topography of the region and it was also used to extract the regions at altitudes of 1500–2500 meters, 2500 to 4570, >2500 and >4570.

### 2.4 Field survey

The impact of climate variability on crop production of a region has been studied by observing local perceptions in different regions of the globe [30, 31]. In the present study, we also visited different sites for verification of change on the ground and recorded narratives of local farmers on the changes. Several rounds of purposive surveys were conducted in Rampur, Shimla (Shimla district, ~ Elevation 1500–2700 m), Bharmour (Chamba district, ~ Elevation 2200–3200 m) and Leo (Kinnaur district, ~Elevation 3500–4500 m) and Kullu and Manali (Elevation ~2000–2300 m) in 2019. We used targeted purposive sampling methods to choose 100 apple growers, 20 each from 5 target areas (Rampur/Shimla, Leo, Bharmour, Kullu and Manali), who were local inhabitants since many years and having apple orchards. Samples were collected through questionnaire with informed consent of the participants.

The intensive field investigations were conducted in 2019. Present study complies all the relevant regulations. All the 100 participants had given informed consent and to maintain high standard of research ethics we did not collect any personal information and their names are anonymous. No child or any vulnerable groups/individuals take part in this survey. Our purpose was to verify the report of apple cultivation from higher altitudes of the Himachal Pradesh solely for academic purpose without harming any living or non-living creatures (S1 File).

## 3. Results

### 3.1 Vulnerable chilling hours

Our calculations show that the number of chilling hours is optimum in the high and mid altitudinal districts compared to low altitudinal ones. The slope value of the chilling hours depicts the number of chilling hours is decreasing (Table 1) in the low altitudinal districts with high intensity since 1975 and mean MK-Stat value for high altitudinal districts is comparatively less than the combined mean MK-Stat value of low and mid altitudinal districts. In the long term, continuous decline in the number of chilling hours may broaden the window for apple cultivations in higher altitudes, which were not favourable for the apple orchards owing to the very high number of chilling hours.

**Table 1:** Calculated chilling hours during DJF at the district level. Value for a district represents the average chilling hours within the whole district and standard deviation(SD) of chilling hours.

Districts	1975–79	1980–84	1985–89	1990–94	1995–99	2000–04	2005–09	2009–14	SD	MK-Stat	Slope
**Chamba**	1722	1776	1771	1766	1771	1763	1761	1748	16	-12^#^	-2.5^#^
**Kangra**	1397	1422	1413	1405	1416	1401	1398	1397	9	-10^+^	-2.1^+^
**Kinnaur**	1191	1196	1174	1171	1184	1165	1154	1175	13	-14^#^	-3.7^#^
**Kullu**	1291	1333	1313	1309	1322	1306	1296	1278	16	-12^#^	-4.4^#^
**Lahul & Spiti**	1525	1644	1634	1629	1640	1627	1622	1611	36	-10^+^	-3.6^+^
**Mandi**	1190	1209	1191	1186	1199	1182	1174	1159	14	-18	-4.7
**Shimla**	1076	1066	1047	1042	1055	1038	1033	1035	14	-22	-5.2
**Sirmaur**	921	899	879	873	888	871	864	877	17	-18	-5.9
**Solan**	1087	1060	1042	1036	1051	1033	1027	1035	18	-20	-6.1

Mann-Kendall and Sen’s slope trend test results are significant at 99%, #significant at 90% and + significant at 80% confidence level.

In two high altitudinal districts (Chamba and Lahul & Spiti), chilling hours were much above the optimum limit due to naturally persisting cool surface temperature caused by the adiabatic process. At the same time, a low but continuous rate of decrease in low altitudinal districts especially in Sirmaur and Solan has directly affected the area and production of apple due to the low number of chilling hours. Their locations being more to the south, geographically has become another factor for declining production based on the current trend, most of the southern/low altitudinal districts are not keeping the minimum threshold number of chilling hours.

The districts of Sirmaur and Solan, have observed a continuous decrease with comparatively very high slope value. These two districts will be on the verge of being unsuitable for apple cultivation and Sirmaur has already entered the league with the least district-average-chilling-hours (Table 1 and Fig 2B). Chamba and Lahaul & Spiti seems improving on the required number of chilling hours since 1975–79 and both the districts are observing decline in number of chilling hours, any further decrease in chilling hours may provide new suitable land for apple cultivation (Table 1)

### 3.2 Relationship of climatic variables with apple area and production

**Fig 3:**
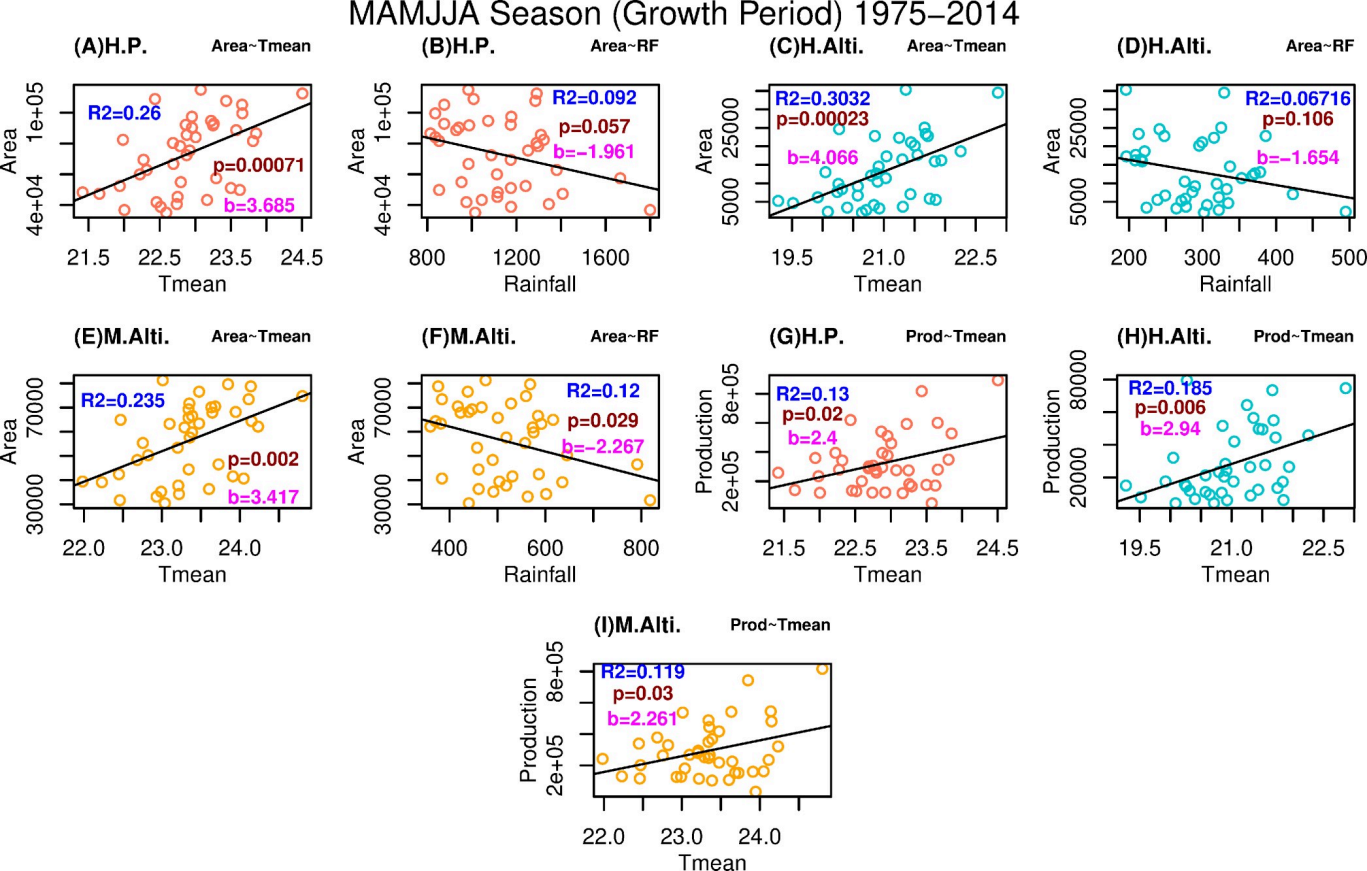
Linear regression based significant relationship of the temperature and rainfall with apple cultivation area and production for growth period season. Relationship trend represented are significant at 90% level of confidence. Red, blue and yellow colour of scatter plot represent the plot for Himachal Pradesh, high altitudinal region, and low altitudinal region respectively. Value of R2, significance level (p), and slope (b) is represented for all the plots with uniformity in colour across all figures.

**Fig 4:**
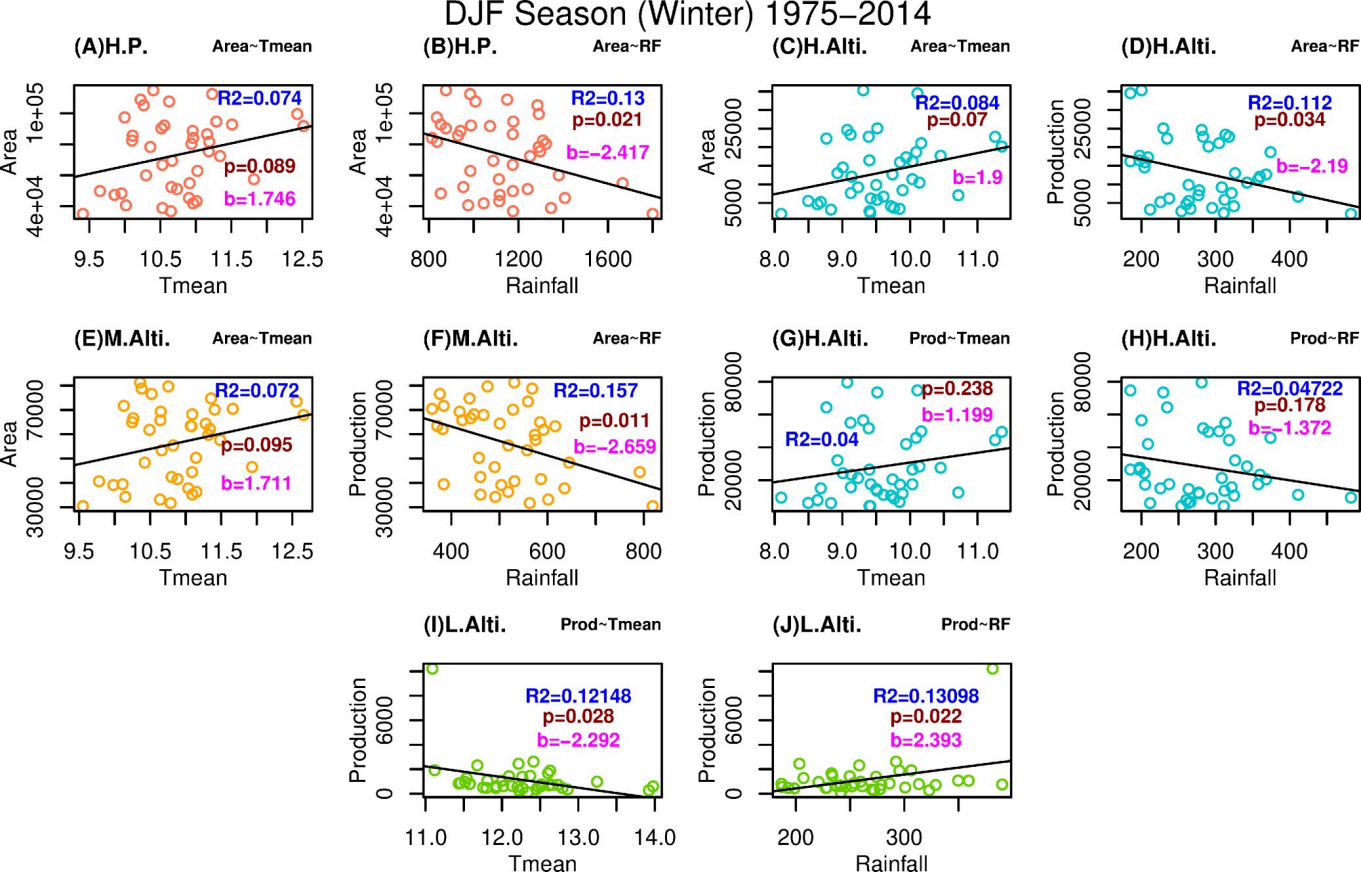
Linear regression based significant relationship of the temperature and rainfall with apple cultivation area and production for growth winter season. Relationship trend represented are significant at 90% level of confidence except panel (G) and (H). Red, blue, yellow and green colour of scatter plot represent the plot for Himachal Pradesh, high altitudinal region, mid altitudinal region and low altitudinal region respectively. Value of R2, significance level (p), and slope (b) is represented for all the plots with uniformity in colour across all figures.

The shifting of apple cultivation towards the higher altitude is associated with shift in the climate of the region. Therefore, the relationship of climatic variables with apple cultivation area and production is analysed here using linear regression technique during growth period and the winter season. Significant regression analysis results with respect to production and climate variables of growth period are found not only for different altitudinal regions but for Pan-Himachal Pradesh (Figs 3 and 4). For the growth period, relationship between area and temperature (area and rainfall) at Pan-Himachal Pradesh level is directly (inversely) proportional to each other as seen in Fig 3A (Fig 3B). Similar relationships are also found for high altitudinal region (Fig 3C and Fig 3D) and mid altitudinal region (Fig 3E and Fig 3F). Low altitudinal districts of Sirmaur and Solan results are not significant (figure not shown). Higher regression slopes in area-temperature and production-temperature plots of high altitudinal region (Fig 3C and Fig 3H) clearly demonstrate the potential increase of apple growing area and production in that region.

Similar trend is observed during the winter season. All the trends of relationship for area and climate variables are having significance of at least 90% level for Fig 4A–Fig 4F. Directly and inversely proportional relationships are found for area-temperature and area-rainfall regressions respectively. Fig 4G and Fig 4H supports that higher temperature will lead to higher production and cultivation area in the high altitudinal region though the relationships are not as significant as seen in Fig 4A–4H. Nevertheless, highly significant relationship of production with climate variables in low altitudinal region is remarkable and therefore winter temperature and rainfall have greater impact on growth period of low altitude productions (Fig 4I and Fig 4J). This significant trend for the low altitude region highlights the edge position of low altitudinal region.

Higher temperature during winter season is a negative factor for apple cultivators there. It is also interesting to find that relationship of temperature with apple production area in mid altitudinal region is not significant at 90% level. It strengthens the point that the production and the area of production are getting stagnate. Particular trend of climate variables and areas of apple production are discussed in the sub-section 3.2.1 and 3.2.2.

#### 3.2.1 Temperature and rainfall trend

**Fig 5:**
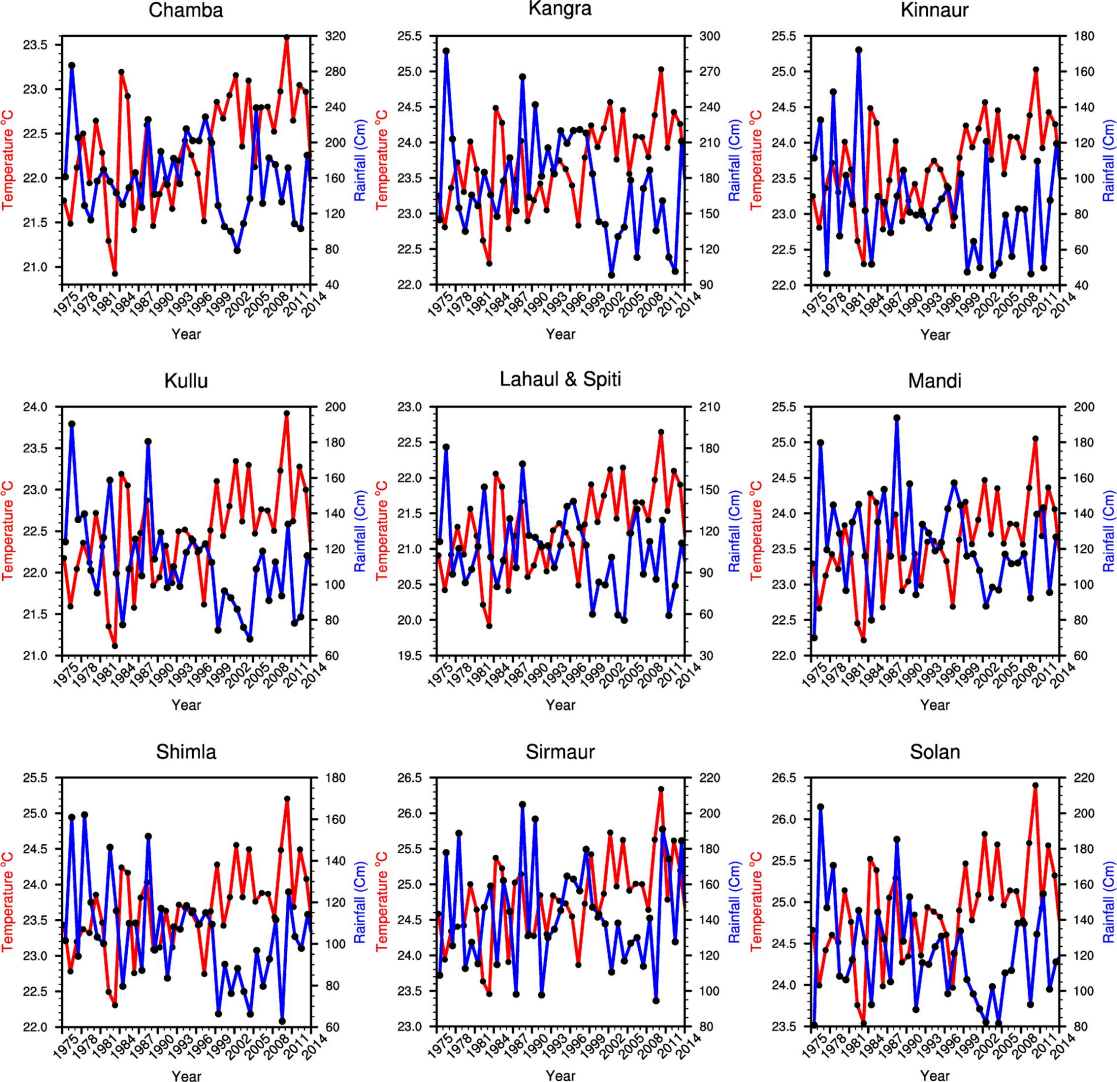
District wise trend of mean temperature for MAMJJA season and total annual rainfall. Name of the district for each graph is represented as the heading of each linear graph. Mean temperature and rainfall are shown red and blue colour solid lines respectively.

An increasing trend in the mean and minimum temperature is found during apple growth period of March to August (hereafter MAMJJA) for the analysis period of 1975–2014. The positive trend (Fig 5) for the period across all the districts indicates a homogenous increase in the temperature for the whole region (Table 2). All the values are statistically significant at 99%, 95% and 90% as indicated in the Table 2. The increasing trend is particularly notable during 2000–2014 as all the districts (Table 3) in the state observed a higher degree of mean temperature anomaly in the MAMJJA as compared to 1975–2014 (Fig 5). In Himachal Pradesh, a rise in the surface temperature has been observed during MAMJJA season as well as DJF season of apple farming.

**Table 2:** District wise trend and significance level of temperature and rainfall obtained using Mann-Kendall test and Sen’s slope for 1975–2014.

Districts/Altitudinal Region	Temperature								Rain (Annual)	
	Max (MAMJJA)		Mean (MAMJJA)		Min (MAMJJA)		Min			
							(DJF)			
	Slope	MK-Stat	Slope	MK-Stat	Slope	MK-Stat	Slope	MK-Stat	Slope	MK-Stat
Chamba	0.03	304	0.03	300	0.02	268	0.02^#^	160^#^	*-0*.*81*	*-104*
Kangra	0.03	292	0.03	296	0.02	258	0.02^#^	162^#^	*-0*.*98*	*-134*
Kinnaur	0.02*	184*	0.02	286	0.03	352	0.03	244	*-0*.*48*	*-124*
Kullu	0.02	266	0.02	266	0.03	318	0.03*	212*	-0.90	-246
Lahul & Spiti	0.03	292	0.03	306	0.03	304	0.03*	222*	*0*.*20*	*-112*
Mandi	0.02	254	0.02	298	0.03	310	0.02*	196*	*0*.*22*	*-106*
Shimla	0.02*	188*	0.02	268	0.03	348	0.02*	220*	0.03*	-186*
Sirmaur	0.02*	186*	0.02	258	0.03	340	0.03	224	0.03*	-186*
Solan	0.01*	210*	0.02	270	0.03	318	0.02*	206*	*0*.*22*	*-106*
1500–2700	0.02	276	0.02	296	0.03	312	0.02*	194*	-0.69*	-200*
2700–4570	0.03	278	0.03	306	0.03	306	0.03*	206*	-0.65	-128
>2700	0.03	276	0.03	304	0.03	308	0.03*	214*	-0.51	-116
>4570	0.03	274	0.03	302	0.03	320	0.03*	216*	-0.42	-98

All values for temperature are significant at 99%

* 95% and

^#^90% confidence level and values in italics for rainfall are not significant.

**Table 3:** District wise anomaly, showing the change in values of climate variables between 2000–2014 (Base period 1951–2000). DJF seasonal anomaly value for 2000–2014 is based on period 1952–2014 because December month is from the year 1951 and January and February are from 1952. Altitude wise change in values of climate variables between 2000–2014 is based on the base period 1951–2000.

**Climate Variable**			**Districts**											
			**Chamba**	**Kangra**	**Kinnaur**		**Kullu**	**Lahul & Spiti**		**Mandi**	**Shimla**	**Sirmaur**		**Solan**
**Rainfall (Annual)**			-28.63	-44.98	-6.99		-20.52	-15.46		-17.97	-18.48	-19.65		-15.93
** Temperature**	**MAMJJA**	**Max**	0.65	0.6	0.32		0.46	0.52		0.47	0.36	0.34		0.41
		**Mean**	0.89	0.51	0.42		0.46	0.48		0.47	0.42	0.4		0.44
		**Min**	0.43	0.44	0.54		0.48	0.47		0.48	0.5	0.48		0.48
	** DJF**	**Max**	0.43	0.3	0.06		0.17	0.3		0.13	0.03	-0.01		0.02
		**Min**	0.39	0.38	0.49		0.42	0.45		0.39	0.43	0.45		0.42
**Climate Variable**			**Altitudinal Regions**											
			**1500–2700**			**2700–4570**			**>2700**				**>4570**	
**Rainfall (Annual)**			-23.3			-17.69			-15.65				-13.59	
**Temperature**	** MAMJJA**	**Max**	0.46			0.49			0.48				0.48	
		**Mean**	0.46			0.47			0.47				0.47	
		**Min**	0.48			0.48			0.48				0.48	
	** DJF**	**Max**	0.15			0.23			0.24				0.24	
		**Min**	0.41			0.43			0.44				0.46	

The pattern of increase across the state remained more or less similar, but the intensity varied latitudinally (district-wise) as well as altitudinally. Hence, the rate of change is analysed at both the dimensions. An increase in surface temperature during the MAMJJA season affects the growth of apple and an increase in DJF season directly influence the number of chilling hours. During the MAMJJA season district-wise analysis shows that the rise in mean and maximum surface temperature is highest in Chamba with 0.89°C and 0.65°C respectively, followed by Kangra, Lahaul & Spiti and Mandi respectively. It is notable here that this change came within a span of only 15 years (2000–2014).

**Fig 6:**
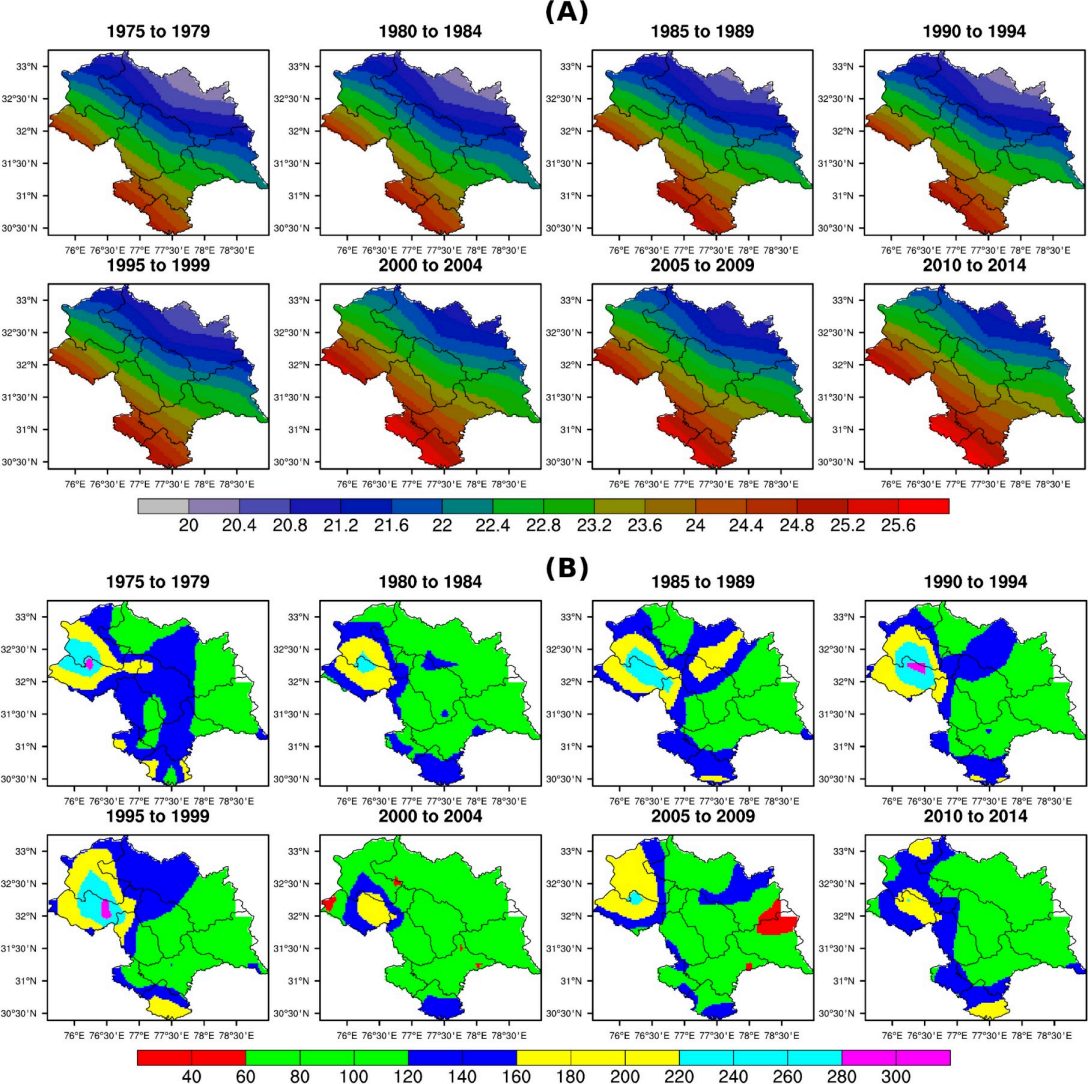
Panel (A) Pentad maps showing the spatial distributions of mean temperature for MAMJJA season from 1975–1979 to 2010–2014; Panel (B) Pentad maps showing the spatial distributions of total annual rainfall from 1975–1979 to 2010–2014.

As shown in Fig 5, districts at higher altitudes are benefiting more from the increasing temperature as extremely cold places are now falling within the optimum temperature threshold for apple cultivation. The gradual shift in the spatial pattern of mean surface temperature towards high altitude is very clear from the pentad map (Fig 6A). An increase in maximum and minimum temperature is also important for apple production. Here it is found that districts with the highest rise in mean and maximum temperature had the least escalation in minimum temperature during apple growth period (MAMJJA) though the rise in minimum temperature is substantial. During winter season (DJF), the increase in the minimum temperature is comparatively higher than the maximum temperature in all the districts. As a result of this increasing winter temperature, number of chilling hours have diminished as they persist only during the winter season.

North Western and Central parts of the state, which have comparatively high altitudes are especially affected by this increase in winter temperature and especially the minimum temperature (Table 1). Furthermore, this increase in the minimum temperature of the winter season (DJF) is found in all the districts (Table 3). Therefore, its role in the shift of the apple orchard is evidently higher than that of the maximum temperature.

Table 3 shows that during 2000–2014 the rate of increase in maximum, minimum and mean temperature is high during MAMJJA in the northern high altitude districts of the state. This is also alarming for the region producing apples at low altitude as the low altitudinal districts are already at the brink of losing optimum temperature threshold conditions during the growth period. It also becomes clear from Table 3 that high altitudinal region at >2700 meter height observed higher increase in maximum and mean temperature as compared to the region between 1500–2700 meter, but the minimum temperature remained almost the same across different altitudes. Therefore, as observed at both the spatial dimensions (i.e. latitudinal and altitudinal), comparatively high increase of maximum temperature during growth period has provided an optimum temperature window for apple production at higher altitudes. Hence, this is one of the common reasons behind the decreasing cultivation area and production of apple in the southern districts at lower altitudes (Table 4).

**Table 4:** Change in apple production and the total area covered during different periods (Base period 1975–2000). The unit of production and area is metric tonnes and hectare respectively.

**District Wise Production**									
**Time Period**	**Chamba**	**Kangra**	**Kinnaur**	**Kullu**	**Lahul & Spiti**	**Mandi**	**Shimla**	**Solan**	**Sirmaur**
**2000–2014**	188.26	90.27	259.39	76.57	507.97	179.56	107.03	-79.2	-46.15
**2000–2007**	135.65	179.98	177.1	82.22	398.66	168.42	63.82	-71.18	-56.99
**2008–2014**	248.38	-12.25	353.43	70.11	632.89	192.29	156.42	-88.37	-33.77
**District Wise Area**									
**Time Period**	**Chamba**	**Kangra**	**Kinnaur**	**Kullu**	**Lahul & Spiti**	**Mandi**	**Shimla**	**Solan**	**Sirmaur**
**2000–2014**	176.2	-13.88	147.08	64.41	1389.87	61.95	35.11	-67.98	-0.89
**2000–2007**	151.25	-10.03	121.02	51.42	251.62	55.14	26.04	-54.54	6.01
**2008–2014**	204.72	-18.28	176.87	79.26	2690.72	69.73	45.47	-83.35	-8.77

Rainfall in all the districts declined over the period 2000–2014 but the loss in the production is seen in the regions where rainfall went out of the optimum threshold limit (100–125 cm) for apple growth. More or less, the rainfall in eastern Himachal Pradesh followed previously present homogeneous pattern (Fig 6B). But the districts located in the south are most affected even with a small decline in rainfall because continuous rise in temperature and decline in rainfall made the already fragile region more vulnerable to climate change. Altitudinally, it is found that the decrease in rainfall is lesser at higher altitudes in Himachal Pradesh. Therefore, apple production in the lower altitudinal regions/districts as shown in Table 3 and Table 4 is to become more vulnerable to climate change impacts.

#### 3.2.2 Changing apple production and cultivated area

**Fig 7:**
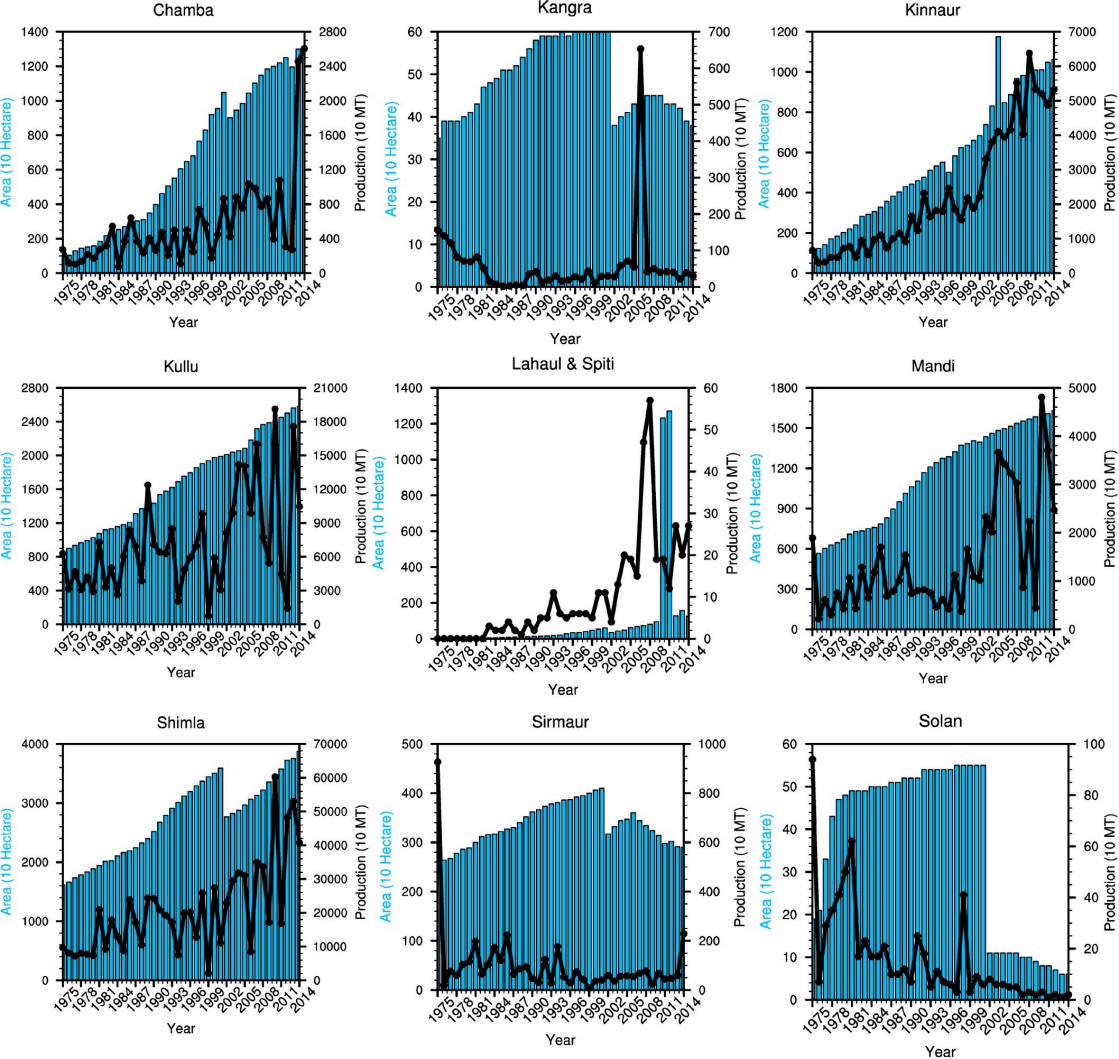
District wise annual trend of area and production (1975–2014). The solid black line shows the production and blue bars represent the total area cover under apple orchards. Here MT is used for a metric tonne of production.

Gradual but intense changes in temperature and rainfall were found to affect the cultivation area and production of apple in Himachal Pradesh. As observed in the high altitudinal region of the state, the temperature increased intensely in the last 15 years (Table 3). The trend shift happened in the year 2000 after which the decline in production is visible in most of the districts and a more pronounced decline is visible in the southern districts that were under apple cultivation earlier (Fig 7). Therefore, changes in climate variables are carefully scrutinized here from the year 2000 onwards.

While discussing the period after the year 2000, it is notable that the declining trend of production and area of apple for the period 1975–2014 is similar (Table 5). The only exception being the trend of the Apple area in Sirmaur, which kept on increasing till 2000 (Fig 7). A long-term average since 2000 (2000–2014) shows decreasing apple production in Solan and Sirmaur districts (both are southern/low altitudinal districts). In addition, Kangra district shows production decline in last 7 years (2008–2014) and area under apple cultivation has declined in all these three districts i.e. Solan, Sirmaur, and Kangra. It is to be noted that Solan, Sirmaur, and Kangra are having low altitudinal extent and these three also have comparatively less area under apple cultivation as compared to their total land coverage. Solan and Sirmaur districts being at the lower latitude and altitude are the most vulnerable to warming of surface temperature.

**Table 5:** District wise trend and significance level value of area covered and production of apple. Slope and MK-Test result is obtained using Mann Kendall’s test and Sen’s slope on the data for the period 1975–2014.

District	Area		Production	
	1975–2014		1975–2014	
	Slope	MK-stat	Slope	MK-stat
Chamba	351.5	758	215.7	392
Kangra	1.0	111	*-2*.*0*	*-23*
Kinnaur	239.3	756	1279.3	660
Kullu	457.3	780	1666.5	240
Lahul & Spiti	27.3	746	5.8	622
Mandi	301.2	778	435.8	312
Shimla	563.8	658	7751.9	380
Solan	-3.5	-62	-7.0	-540
Sirmaur	22.8	230	-13.7^#^	-164^#^

All value for area and production are significant at 99% confidence level, * significant at 95% confidence level

^#^ significant at 90% confidence level and value in italics for rainfall are not significant.

It is also noted that Mandi, which is at a slightly higher altitude (within the range of 1500–2700 meters) with a larger cultivation area, has been the next most vulnerable district. The Cultivation area and apple productivity in Mandi are stagnated. A negative growth is soon expected as it is the 4^th^ district in the list that has a comparatively high maximum temperature recorded during the growth period in addition to the decline in rainfall amount. Middle altitudinal districts i.e. Kullu, Kangra, and Mandi are also stagnating with respect to production, however districts at the lower altitude (Sirmaur and Solan) are facing negative trajectory in production (except Shimla) and area of cultivation.

**Fig 8:**
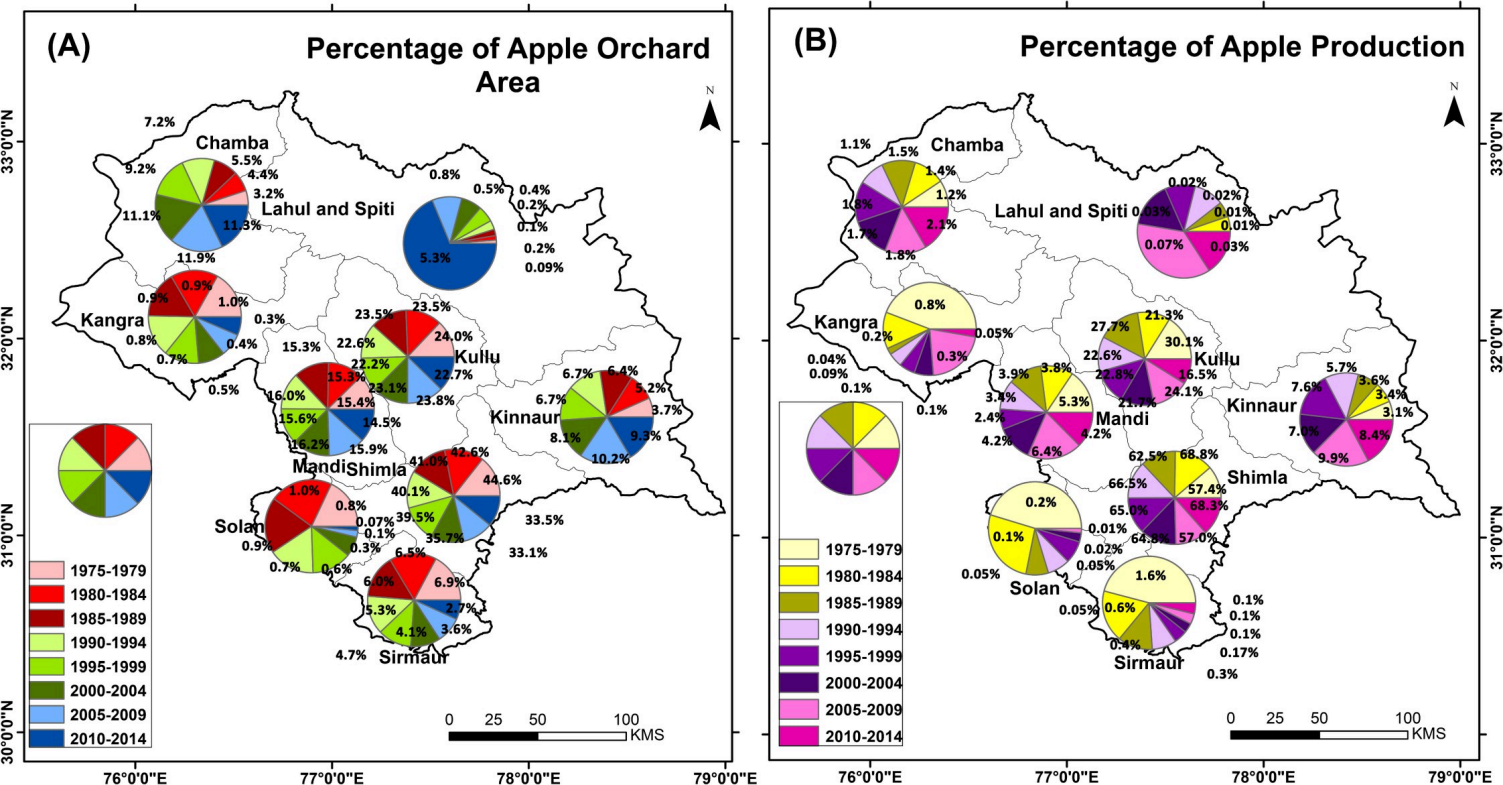
Panel (A) District wise percentage of area covered under apple orchard in Himachal Pradesh. Panel (B) District wise percentage of apple production in Himachal Pradesh. The percentage is given in panel (A) and panel (B) is in respect of the total area of an apple orchard in Himachal Pradesh and total production of apple in Himachal Pradesh respectively. Here, the pie chart’s covered area is for periodic visual comparison at district level.

**Fig 9:**
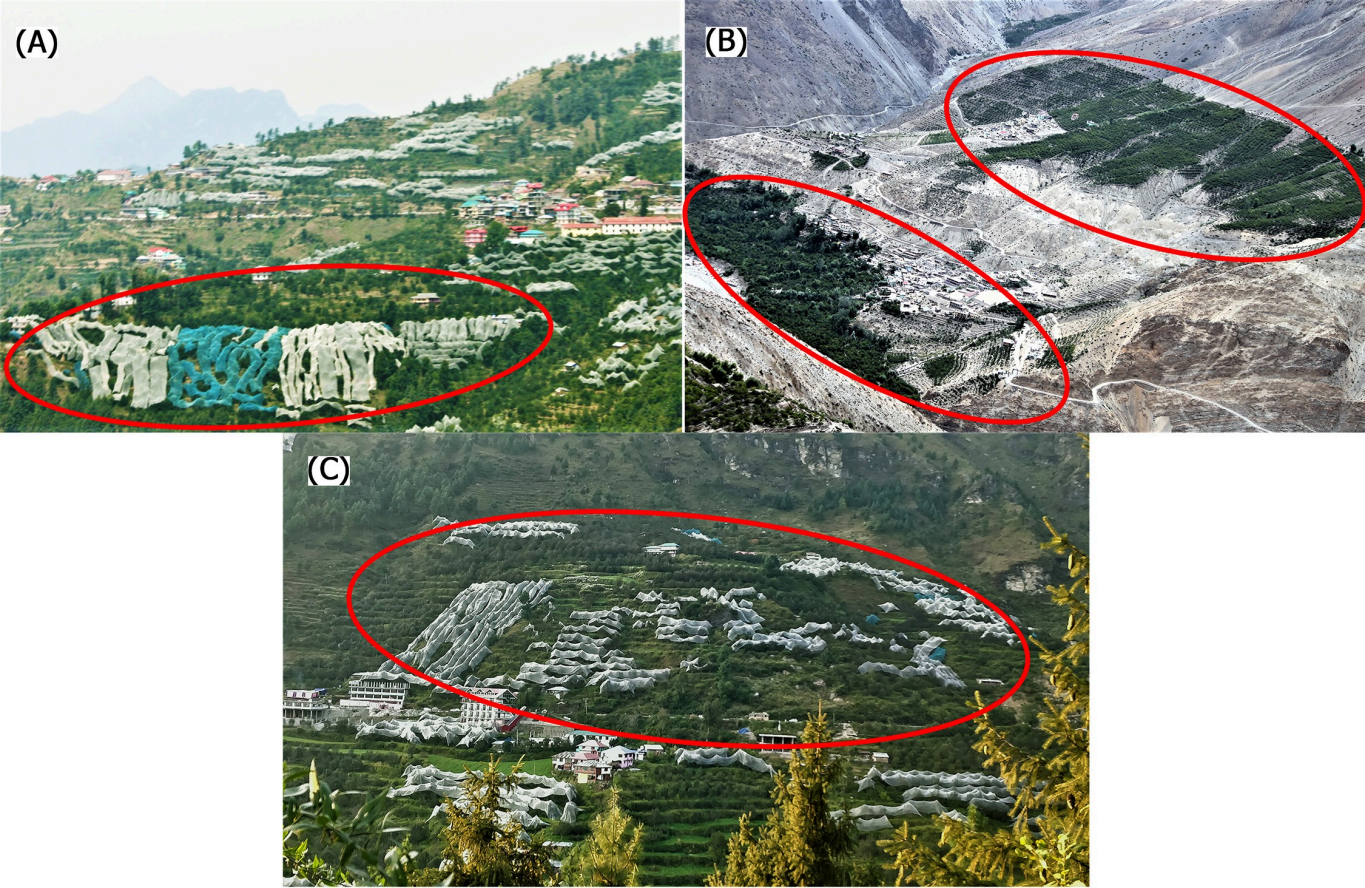
Sample photographs collected as part of the primary survey in the year 2018–2019. Panel (A) Picture of Rampur in Shimla district at an altitude of 2200–2700 m; Panel (B) Leo village at altitude of 3200–4500 m. in Kinnaur district with the apple orchard highlighted using red ovals; Panel (C) Near Manali in Kullu district at the altitude of 1500 m.

During 2000–2014 some of the other districts in the region have seen an increase in the production and cultivation area. For example, Chamba district has observed the increase in the cultivation area. Interestingly, this district has also observed highest increase in maximum temperature during the growth period (highest increase in mean temperature of 0.89°C) as well as the winter months. That rise in maximum temperature was greatly compensated by the fact that most cultivated areas of the district are located to the north (latitudinally higher regions) and higher altitudes. Similar conditions are also found for Lahaul & Spiti district. Altogether, Chamba, Lahaul & Spiti and Kinnaur (High latitudinal and altitudinal districts) have the positive percentage trajectory for the area of cultivation (Fig 8A) and production (Fig 8B). The positive trend found from the sites visited during the primary survey in the high altitudinal regions of the state is also corroborated by maps of google earth (Fig 9A and 9B).

### 3.3 Socio-economic survey

In Rampur of Shimla district, apple orchards already shifted to uphill regions and cultivation of apple is shown in (Fig 9A). If the rate of upward shift will continue to maintain the optimum conditions for apple growth, apple orchards will vanish in many areas within 10–15 years as has been the fate of Solan and Sirmaur. On the other hand, the expansion of apple orchard is seen in Leo village of Kinnaur district (Fig 9B) and in Manali of Kullu districts at the uphill (Fig 9C).

The shift in apple orchards to Kinnaur is associated with the decrease in snow depth. 71 years old anonymous participant of Leo village, who grows apple since 2002, said ‘there is a decrease in snow depth from 4–5 fts in early 1970s to 1–2 fts during 2017–18’. Another 51 years old anonymous participant from Maling told that during winter months they were totally cut off for 4–5 months in 1970-80s due to dense snow and even not able to move to Nako village situated at 5 kms. But now a days only 1–2 months are with snow but with much lower depth than what they have seen during their childhood that indicates decrease in snow depth in higher altitudes may be influenced due to the observed hottest decades of the earth recorded during the last decades. Even if there are mountains and valleys that have local topographic ups and downs influencing the spatial pattern of the temperature, our field observation, survey from ground and farmers’ observations reveals that apple plantations are possible at higher altitudes of the Himachal Pradesh.

**Fig 10:**
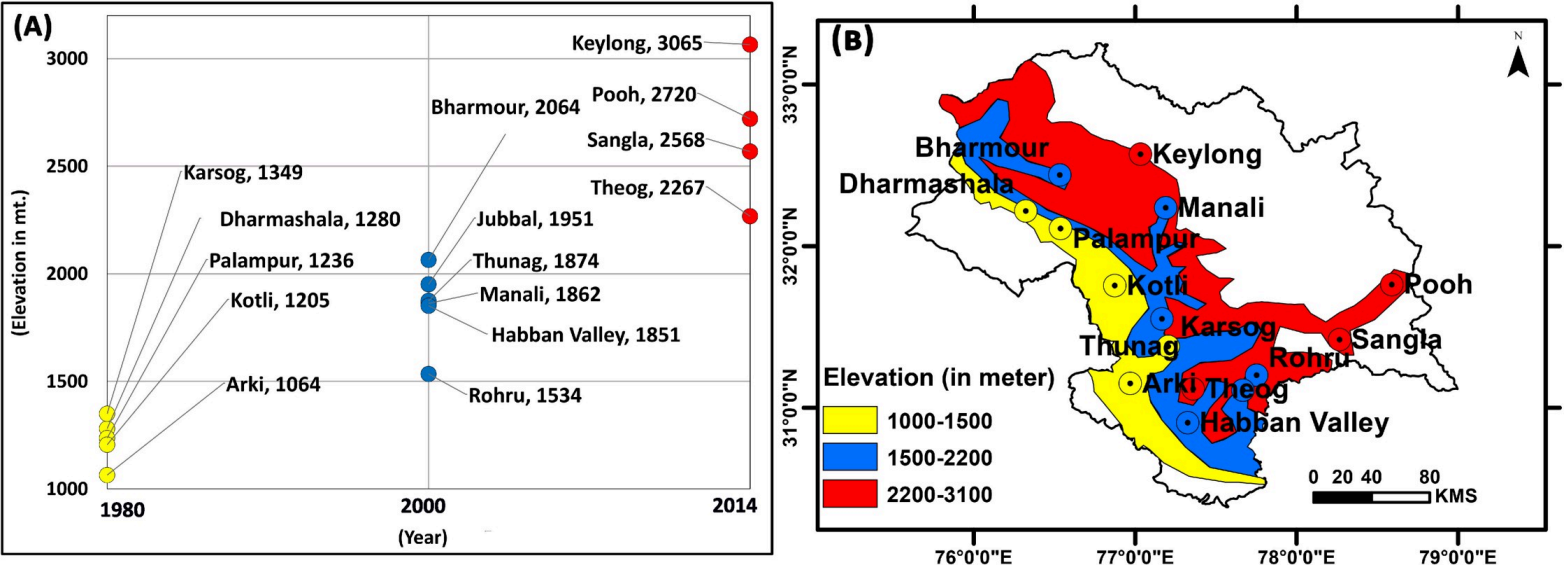
Panel (A) Temporal shift of the apple orchards at different period. Yellow, blue and red points are for years 1980, 2000 and 2014 respectively. Panel (B) Spatial representations of the sites growing apple at different altitudes in Himachal Pradesh.

During 1980s apple were grown within 1200–1500 m elevations in Arki, Kotli, Palampur, Dhrmashala and Karsog of Himachal Pradesh, by 2000 it shifted to 1500–2500 m elevation of Rohru, Habban valley, Manali, Thung, Jubabal and Bharmour, and by 2018–19 apples are grown in 2500–3500 m elevations in Theog, Sangla, Pooh and Keylong areas of higher mountains regions (Fig 10A and Fig 10B). Apples productions in lower altitudinal areas are now considered of very low quality whereas quality apples in Kinnaur fetch a good price for the farmers.

During our field investigations we also observed a shift in crop pattern. In the lower altitudinal areas farmers are now cultivating peas, potatoes, plumb whereas in higher altitudinal areas farmers started growing apples instead of peas and potatoes. This shift in cropping pattern influenced their economy and livelihood. 90% of respondents said a decrease in snow depth that influenced the chilling hours during winter is the main reason behind this shift in apple orchards. Decrease in snow depth were very well observed in previous studies of the Western Himalayas including Himachal Pradesh [32–34].

## 4. Discussion

This study suggests that the shift of apple orchards towards the high altitudinal region in the Himachal Pradesh of India is due to continuously decreasing chilling hours for the whole state (especially after the year 2000). Transformation of high altitudinal regions (especially Lahaul & Spiti) into the suitable region of apple cultivation is also supported and discussed by Rana et al. (2011) [35]. The chilling hours during the DJF season contribute the most to the apple production and a rapid increase in the minimum temperature during this season has created an unfavourable condition by reducing the number of chilling hours. This trend has also been observed in different observing stations in the past. For example, in a station based study [36], an experiment was conducted in controlled conditions to find out the importance of the contribution of a varying number of chilling hours. It was found that flowering did not take place in the absence of optimum number of chilling hours. There are several such studies that clearly established the fact that optimum chilling hours and temperature during the growth period play a very crucial role in apple production.

The calculated chill units using station data trend in our results is in agreement with that of some other studies for apple cultivation in Himachal Pradesh. For example, Chand et al. 2016 and Sen et al. 2015 used the UTAH model to calculate the chill units of a station in Kullu district and found a similar decreasing trend as presented in the present study [37, 38]. But, the use of Dale. E Linvill’s Modified UTAH Model is of significance for the present study area due to differential temperature weights and no enhancement by moderate temperature prevalence. Pros and Cons and application of the Dale. E Linvill’s Modified UTAH Model in various studies have been discussed in detail by Luedeling E. (2012) [39].

Along with chilling hours, change in temperature also leads to stunted growth, diseases and ultimately the quality of the apple crop. A study on different regions of China has made it clear that change in temperature leads to the deterioration of the quality of apple crop [40] and following the law of nature similar conditions leads to low quality / low production of apple in Himachal Pradesh. The impact of changing temperature has also been noted by Kumar et al. (2009) in the productivity of cabbage seeds in north western Himalaya [41]. Moreover, scarcity of rainfall has been a problem and it is compensated by the irrigation through various means.

Soil pH level is also one important condition but as it is found within optimum across Himachal Pradesh, it was not considered in this study. However, except all the above required farming conditions, snowfall in the state is also considered as an important factor. But, there were some difficulties in completing snowfall based analysis; one of the important factors was the non- availability of the gridded snowfall data with high resolution. Only station based snowfall data was available for a few stations with irregular time periods. Therefore, to fulfil the requirement of completing this important study, we adopted another important and scientific method of calculating the chilling hours.

As far as the temperature is concerned, as an independent parameter, mainly minimum temperature in DJF season and maximum temperature in MAMJJA (growth period) have brought Chamba and Lahaul & Spiti in the group of districts of positive growth in apple production and cultivated area. On the contrary, Kangra, Kullu and Mandi being middle districts are the one with good share of low altitudinal area and having larger impact on apple due to rise in maximum temperature during MAMJJA. Increasing temperature in the region has affected the southern districts (Sirmaur and Solan) strongly, middle districts (Kangra, Kullu Shimla and Mandi) in moderately and northern districts (Chamba, Lahaul & Spiti and Kinnaur) into favourably.

Impact on the apple orchards has been due to the changing climatic conditions but primary survey in different sites in the state reveals that consistent good income from the apple production is a good option for financial security hence a major encouraging force behind the apple cultivation. In the present scenario, farmers in the northern districts are benefiting while the others in southern low altitude districts are adversely affected. Up in the high altitudinal districts, recent onset of the optimum climate conditions is appealing factor for adopting apple cultivation as another option for income generation.

Thus, continuous warming of temperature in the region will definitely benefit (harm) regions with high altitude (low altitude). People in the low altitudinal region, who used to depend only on apple crop for their livelihood are now compelled by the adverse climatic conditions to shift towards the vegetable crops e.g. peas, potato and other vegetable crops. These vegetable crops in lower altitudes do not generate income at par with apple crop to compensate the loss and therefore many male members are migrating to urban areas within the state or somewhere in distant areas of the country for income (observation from the primary survey). Apple crop was the main factor for comparatively high living standard of the people and therefore loss of expected income in the present scenario will take away some of their comforts.

## 5. Conclusion

Based on the present study, it became evident that apple was not cultivated in Leo of upper Kinnaur, Bharmour of Chamba, and Keylong of Lahul and Spiti during early 1980s. Farmers in these high lands of Himachal Pradesh have started growing apple in early 2000s on a trial basis and by 2015 contributions from apple productions have hugely contributed to their livelihood. However, this shifting in apple cultivations while making farmers in the higher altitudes richer made farmers in lower altitudes of Solan and Sirmaur becomes poorer due to consistent reduction in shape, size and quality of the apples in those areas. Hence, these farmers shifted their cultivation pattern by adopting the farming of peas, potatoes and plumb in these lower altitudes areas.

The spatio-temporal agro-climatic impact in the region is getting affected or is going to get affected due to warming of temperature and decrease in rainfall. Here, it is found that major factors for the apple production shift in cultivation areas were chilling hours, that persist mostly in the winter months, and the surface temperature during growth period. However, some other factors can play their roles to impact various crops. Therefore, there is a lot of potential in finding out the potential impact for various other major crops in the region and as a consequence of it, policies can be framed to mitigate it well in time to save the security of livelihood of people in the region. Also it needs to be seen how far these shifts can move in near future in the climate change scenarios.

## Supporting information

S1 File (DOCX)
