# Supplementary material for: Why apple orchards are shifting to the higher altitudes of the Himalayas?
Source: PLoS One. 2020 Jul 10;15(7):e0235041. doi: 10.1371/journal.pone.0235041 (PMC7351220; doi:10.1371/journal.pone.0235041)
Supplement: S1 File — (DOCX) [file pone.0235041.s001.docx]

Why Apple Orchards are Shifting to the Higher Altitudes of the Himalayas?

Netrananda Sahu^1*2^, Atul Saini^1^, Swadhin K. Behera^3^, Takahiro Sayama^2^, Limonlisa Sahu^4^ , Van-Thanh-Van Nguyen^5^ ,Kaoru Takara^6^

**QUESTIONNAIRE**

**Why Apple Orchards are Shifting to the Higher Altitudes of the Himalayas?**

**Objectives**

- To identify the shifting of apple orchards in Himachal Pradesh.

**Research Ethics**: Academic and scientific first-hand information and field verification survey has been conducted in Rampur/Shimla, Leo, Bharmour, Kullu and Manali of Himachal Pradesh, India in 2019. All the 100 participants had given informed consent and to maintain high standard of research ethics we did not collect any personal information and their names are anonymous. No child or any vulnerable groups/individuals take part in this survey. Our purpose was to verify the report of apple cultivation from higher altitudes of the Himachal Pradesh solely for academic purpose without harming any living or non-living creatures.

**A. General Information:**

**1. Village:**

|  |
| --- |

**2. Altitude of the Village**

|  |
| --- |

**3. Respondent: Anonymous**

| Serial No: |
| --- |

**4. Age: 21 years above only**

|  |
| --- |

**B. APPLE ORCHARD BASED INFORMATION:**

**5. How long have you been living in this area ?**

|  |
| --- |

|  | Increasing/Decreasing Temperature |  |
| --- | --- | --- |
|  | Low/Heavy Snowfall |  |
|  | Low/High Rainfall |  |
|  | Any Other |  |

**6. Have you observed any change in weather/climate in your locality ?**

**7. When apple cultivation started in your village/locality?**

|  | 10 years before |  |
| --- | --- | --- |
|  | 20 years before |  |
|  | 30 years before |  |
|  | Can’t Say |  |

**8. Before apple, what type of crops you cultivated in your land?**

|  | Mushrooms |  |
| --- | --- | --- |
|  | Peas/potatoes |  |
|  | Fruits |  |
|  | Others |  |

**9. Do you observe any significant change in apple production of your area?**

|  | Increase in Production |  |
| --- | --- | --- |
|  | Decrease in Production |  |
|  | Stagnating Production |  |
|  | Can’t Say |  |

**10. What are the climate factors in shifting apple orchards to the higher**

**altitudes as per your observation?**

|  | Increasing/Decreasing Temperature |  |
| --- | --- | --- |
|  | Low/Heavy Snowfall |  |
|  | Low/High Rainfall |  |
|  | Any Other |  |

**11**. If apple will not cultivate in future like in the case of Solan and Sirmur

What type of crops will you grow?

12. **Have you seen any significant change in the cropping pattern in previous years?**

|  | Very much |  |
| --- | --- | --- |
|  | Significant changes |  |
|  | Not any change |  |
|  | Don’t know |  |
